# The Effectiveness of Health Education in Improving Knowledge about Hypoglycemia and Insulin Pen Use among Outpatients with Type 2 Diabetes Mellitus at a Primary Care Hospital in Vietnam

**DOI:** 10.1155/2021/9921376

**Published:** 2021-08-27

**Authors:** Loan Thi Chu, Tran Que Nguyen, Phuong Thu Thi Pham, Truc Thanh Thai

**Affiliations:** ^1^Faculty of Nursing and Medical Technology, University of Medicine and Pharmacy at Ho Chi Minh City, 217 Hong Bang, District 5, Ho Chi Minh City, Vietnam; ^2^Department of Nursing, University Medical Center Ho Chi Minh City, 215 Hong Bang, District 5, Ho Chi Minh City, Vietnam; ^3^Hospital for Rehabilitation-Occupational Diseases at Ho Chi Minh City, 313 Au Duong Lan Street, District 8, Ho Chi Minh City, Vietnam; ^4^Faculty of Public Health, University of Medicine and Pharmacy at Ho Chi Minh City, 217 Hong Bang, District 5, Ho Chi Minh City, Vietnam

## Abstract

**Background:**

Patients with type 2 diabetes mellitus (T2DM) who have limited knowledge about hypoglycemia and insulin pen use are likely to have hypoglycemia and other complications.

**Objective:**

This study aimed to evaluate the effectiveness of health education on knowledge about hypoglycemia and insulin pen use among outpatients with T2DM at a primary care hospital in Vietnam.

**Methods:**

A pretest–posttest study was conducted among 80 patients with T2DM at District 11 Hospital in Ho Chi Minh City, Vietnam. At baseline, patients were interviewed through a predefined, structural questionnaire to assess their knowledge about hypoglycemia and insulin pen use. After that, patients underwent an individual health education session about hypoglycemia and insulin pen. One month and two months after this intervention, knowledge about hypoglycemia and insulin pen use were recorded again.

**Results:**

The majority were males (65.0%) and the mean age was 59.6 (standard deviation 8.1, range 35-75) years. Very few patients had good knowledge and proper insulin pen use, with percentages ranging from 13.8% to 60%. There was a significant improvement of knowledge and practice after the intervention. Such improvement remained high one month and two months after the intervention.

**Conclusions:**

The health education intervention is effective in improving knowledge and practice in this population. There is a pressing need for such intervention at primary care hospitals to optimize treatment for patients with T2DM, possibly focusing on those who had characteristics to have the best effectiveness found in this study.

## 1. Introduction

Diabetes mellitus (DM) is becoming a global public health problem, characterized by its high prevalence and mortality. Globally, there were more than 460 million people diagnosed with DM in 2019, which is estimated to rise to 700 million by 2045. The prevalence of DM is higher in low- and middle-income countries with the dominance of type 2 diabetes mellitus (T2DM) [1, 2]. Diabetes mellitus is among the leading causes of deaths worldwide, accounting for 1.6 million deaths each year. It also leads to several severe complications to the heart, kidneys, eyes, nerves, blood vessels, and teeth during the course of the disease [3].

In Vietnam, DM is recognized as a major public health burden with approximately 5.76 million people suffering from this condition. The age-adjusted prevalence doubled from 2.7% to 6% between 2002 and 2017 [4, 5]. Diabetes mellitus is the top cause of mortality and disability combined and represents 3.96% disability-adjusted life years [6, 7]. Coupled with the aging population in Vietnam, the prevalence of negative impacts of DM on individuals and society presents an urgent demand for proper intervention and management strategies.

Besides lifestyle modification and oral antidiabetic medications, glycemic control is the cornerstone of diabetes management strategy [8]. Insulin therapy, which is essential for treating of both type 1 diabetes mellitus and T2DM, plays a vital role in the maintenance of blood glucose level and reduces diabetes complications. Of the variety of insulin being introduced, the insulin pen appears to be easier to use, portable, accurate, and safe compared to traditional vial and syringe [9, 10]. Effective insulin management using an insulin pen helps patients improve adherence, facilitate self-management of people with DM, prevent the risk of hypoglycemia, and improve the quality of life [11, 12].

However, a large body of literature indicates that patients with DM have insufficient knowledge about hypoglycemia [13, 14] and insulin use [15, 16]. The lack of such knowledge will likely result in the increased risk of hypoglycemia and severe complications. Therefore, strategies for enhancing knowledge about hypoglycemia and insulin use in patients with T2DM need to be developed. Among intervention approaches, health education is a key strategy in diabetes management to improve knowledge and practice related to self-management of hypoglycemia and insulin use [17, 18]. However, to date, little is known about the effectiveness of health education in enhancing the knowledge of hypoglycemia and insulin pen use in outpatients with T2DM who manage their condition at home, particularly in settings like Vietnam.

Therefore, this study is aimed to evaluate the effectiveness of health education on knowledge about hypoglycemia and insulin pen use among outpatients with T2DM at a primary care hospital in Vietnam and to examine the potential factors influencing this effectiveness. Findings from this study can serve as scientific evidence for further development of well-designed healthcare programs to optimize the treatment and to improve the quality of care and quality of life in patients with DM.

## 2. Methods

### 2.1. Study Design

During December 2019 and May 2020, a pretest–posttest one-group quasi-experimental study was conducted at District 11 Hospital in Ho Chi Minh City, Vietnam. The hospital is a typical district hospital in Vietnam and serves as a primary care clinic for approximately 350 outpatients with T2DM.

### 2.2. Participants

Outpatients with T2DM aged 18 or more, who had been using insulin pen for at least one month and agreed to participate in this study, were recruited. Patients with comorbidities that affected participation such as those with cognitive impairment were excluded. Patients who could only use insulin pen with help from family members and were unable to use insulin pen on their own were also excluded. Participation was on a voluntary basis.

The sample size calculation was based on the formula to detect the difference in the prevalence of good knowledge and practice before and after the intervention. The estimation for sample size calculation was based on a previous study evaluating the effectiveness of the health education approach in Vietnamese patients with T2DM [19]. With the expected conservative improvement of about 30%, from 40% before the intervention to 70% after the intervention, type one error rate of 5%, a sample size of at least 60 was required to have a statistical power of 90%. In this study, we recruited 84 patients. However, 4 patients refused to participate in the study due to not having enough time for the study.

### 2.3. Study Procedure

Participants were interviewed using a predefined structural questionnaire to measure their knowledge about hypoglycemia and insulin pen. Participants were asked to demonstrate their use of insulin pen on a model and their level of practice was observed and recorded. After that, patients underwent an individual health education session about hypoglycemia and insulin pen. The researcher first presented these two topics using both Microsoft PowerPoint and hard-copy flashcards. Patients also watched a manual video from the manufacturer on using insulin pen based on the pen they used. Patients were offered 15 minutes to practice using insulin pen. A take-home booklet with information about these two topics was also provided to the patients. The measurement of knowledge and practices as described above was conducted again after the health education session. One month and two months after this intervention, knowledge about hypoglycemia and insulin pen use were recorded again. After each interview and observation during the follow-up, patients underwent an individual health education session to reinforce their knowledge and practice.

### 2.4. Measurement

The structural questionnaire included three main parts. The first part was about patients' characteristics including sex, age, ethnicity, education level, occupation, and the average monthly income. Information about health status, such as the duration of living with diabetes, the duration of time using insulin pen, and the number of insulin injections per day was also included. To discriminate the effect of this intervention with others, we included information about health counseling services patients received. The second part had five questions to measure knowledge about hypoglycemia, including definition, symptoms, testing, treatment, and prevention of hypoglycemia based on the standards of medical care in diabetes by American Diabetes Association [20] (Appendix Figure 1). One point was given for a correct answer to each question and the overall score was the total score of all five questions, ranging from 0 to 5. The last part was to measure knowledge about using insulin pen based on the Indian recommendations 2.0 for best practice in insulin injection technique [21]. One point was given for a correct answer to each of the 15 questions included. The overall score was the total score of all questions, ranging from 0 to 15 (Appendix Figure 2).

A 15-step checklist was used to evaluate practice on insulin pen use. The checklist was based on EADSG Guidelines and manuals from the manufacturer and included information about the preparation, attach needle, prime the insulin pen, select insulin pen, inject the insulin, and remove needle [22]. The overall evaluation of practice was based on the total score of this checklist, ranging from 0 to 15, and a higher score indicates better practice (Appendix Figure 3).

The questionnaire and checklist were originally developed in Vietnamese and were sent to 3 experts (i.e., experienced nurses and doctors) to review. These were also tested among 10 patients to double-check the logic and wording. A minor revision was made, mostly in the Vietnamese wording, before the main study.

## 3. Data Analysis

Data were entered into EpiData 3.1, cleaned, and double-checked to ensure no error during data entry. Final data were exported to Stata 16.0 for data analysis. Descriptive statistics used included frequency and percentage for qualitative data. Due to the skewed distribution, scores on knowledge and practice were presented as median and interquartile. The McNemar's Chi-squared tests were used to compare each aspect of knowledge and practice before the intervention and after the intervention. To identify factors associated with the improvement of knowledge and practice after the intervention, Generalized Estimating Equation (GEE) was used. The use of GEE was to consider self-matched, repeated measure nature of outcomes in this study. All statistical tests were two-sided, and the type one error rate was set at 5%.

### 3.1. Ethics Approval

All procedures in this study were approved by the Ethics Committee in Bio-Medical Research at the University of Medicine and Pharmacy at Ho Chi Minh City, Vietnam (518/ĐHYD-HĐĐĐ). Approval was also granted by the Director Board of District 11 hospital. Participation was on a voluntary basis, and written informed consent was obtained from all patients participated in this study.

## 4. Results

Among 80 patients with T2DM who participated in this study, the majority were males (65.0%) and the mean age was 59.6 (standard deviation 8.1, range 35-75) years. Most participants had been living with diabetes for more than 6 years (76.3%), used insulin pens for at least one year (71.2%), and injected insulin at least twice a day (87.5%).

The measurement of knowledge about hypoglycemia is presented in Table 1. Before the intervention, very few patients had good knowledge about definition (13.8%), testing (30.0%), treatment (15.0%), and prevention (11.3%) of hypoglycemia, except hypoglycemia symptoms (60.0%). Knowledge of these aspects was significantly improved right after the intervention. Although the prevalence of good knowledge one month after the intervention decreased slightly, the figures remained high after two months. A similar pattern was observed in the total score. There was a significantly increased trend in the knowledge about hypoglycemia prevention.

Table 2 presents the levels of knowledge about insulin pen among patients with T2DM. A low level of knowledge about insulin pen was observed in most aspects measured before the intervention. However, there was a significant improvement after the intervention. Such improvement remained high one month and two months after the intervention. The highest increase was recorded in knowledge about pushing the air bubble out before injection (from 20% before the intervention to 93% two months after the intervention), the number of injections per needle (10% and 80.3%), consequences of reuse needle so many times (11.3% and 81.7%), and needle treatment after injection (7.5% and 80.3%).

Participants had good practice using insulin pen (Table 3). The ceiling effect was observed in almost half of practice evaluated where patients had good practice before the intervention, and thus, there was no more room for improvement. However, patients had improper practice toward priming the insulin pen with low percentages of good practice in this aspect before the intervention ranging from 7.5% to 13.8%. These figures increased significantly right after the intervention and during the one-month and two-month follow-ups.

The association between patients' characteristics and the overall knowledge and practice scores from all time points were identified using GEE and are presented in Table 4. In overall, significantly higher improvement in knowledge and practice was found among young patients with high monthly income and those who had received counseling about insulin pen since their diagnosis. The high education level was associated with high improvement in knowledge, but not for practice. Interestingly, patients who had received counseling about insulin pen from nurses and doctors since their diagnosis had significantly lower improvement in knowledge about insulin pen compared to those who had received such information from pharmacists.

## 5. Discussion

This study is among the first in Vietnam to evaluate the effectiveness of health education on improving knowledge about hypoglycemia and insulin pen use among outpatients with T2DM at a primary care hospital in Vietnam. The findings highlighted that the levels of knowledge of hypoglycemia and insulin pen use were not optimal at baseline but were significantly improved after the health education intervention. The improvement remained significant after two months.

People with insulin-treated DM are susceptible to hypoglycemia. Therefore, they must have sufficient knowledge to prevent hypoglycemia and to increase effective self-management. This study found that patients had a relatively high level of knowledge about hypoglycemia symptoms, but inadequate knowledge regarding blood glucose level for hypoglycemia, the importance of glycemic test once hypoglycemia occurs, and measures for treatment and prevention of this condition. The total measuring score indicated an overall poor knowledge of hypoglycemia among participants. These findings agree with previous studies that showed good knowledge about hypoglycemia symptoms [13, 23, 24], and poor knowledge about DM and other aspects of hypoglycemia [13, 14]. An effective management of DM and hypoglycemia is not merely based on the recognition of symptoms but also on the awareness of other important components such as causes, complications, glycemic level monitoring, treatment, and prevention for hypoglycemia onset. Notably, only 11.3% of patients in our study had good knowledge about prevention, which is much lower than that reported in previous studies. For example, among 15,892 Japanese patients with DM aged 65 or more, 63% had good knowledge about this aspect [25]. The good knowledge of hypoglycemia prevention was also found in Ethiopia [24].

Significant improvement in knowledge about hypoglycemia was found in our study with a large proportion of participants correctly responded almost all aspects of hypoglycemia at the end of the follow-up, ranging from 80.3% to 90.1%. In line with the effectiveness of health education in improving knowledge of hypoglycemia, in a systematic review, LaManna et al. (2019) [18] indicated the positive impacts of education on hypoglycemia outcomes, regardless of the intervention approaches or delivery (structured questionnaire, diabetes self-management education and support, individual/group sessions), educators (doctor, nurse, pharmacist, others), participants, study design, multifaceted methods, and the length of interventions. A six-month longitudinal study in India also demonstrated a significant improvement in knowledge, attitude of patients with DM, and a decrease in hypoglycemia symptoms and episodes [17]. These studies and ours indicated the important role of health education in enhancing knowledge of hypoglycemia among those with DM over time. However, the levels of knowledge of hypoglycemia after the one-month follow-up in our study decreased slightly compared with those recorded right after the intervention. Several likely explanations for this decrease are (1) patients were likely to forget the information if it was provided just once, (2) they might have underestimated the importance of the hypoglycemia occurrence because they had never had hypoglycemia events, or (3) they overtrusted in their capability of self-management. This finding suggested that a more frequent follow-up and repeatable interventions to remain the effectiveness, especially in the early stage of intervention, can be beneficial.

Literature has shown that good knowledge of insulin use is associated with adherence to insulin therapy, effective blood glucose level control, self-management, and reduction of adverse outcomes [26, 27]. However, in our study, participants only had a good awareness of some aspects of insulin pen use. Participants demonstrated low knowledge in several important steps of the procedure, such as lack of pushing out the air bubbles (20%) and stabilizing pen temperature (32.5%) before each injection, improper storage of used insulin pen (26.3%), and insulin needle reuse (90%). In accordance with the present findings, previous studies showed insufficient knowledge about insulin pen use in patients with T2DM [15, 16, 28]. For example, in a recent nationwide survey in Bangladesh, Kamrul-Hasan et al. (2020) [28] found high rates of pen users reusing needles (98.5%) and reusing them over 10 times (38.9%), possibly due to a lack of awareness of the possible number of injections per needle and the consequences of needle reuse. The repeated use of insulin needles can lead to distortion, bending, breakage, and complications, including pain, bruising, bleeding, infection, and lipohypertrophy [16, 28]. The common error of needle reuse has raised a major concern and, in turn, should capture more attention of healthcare professionals.

Despite the low extent of knowledge about insulin pen, participants in our study had a relatively good insulin injection practice. However, they had errors in some steps of the injection process. Consistent with the literature [29, 30], this study found that patients skipped all aspects of priming insulin pen before injection, which could affect the effectiveness of needle and the existence of air bubbles. It has also been suggested to keep the needle under the skin in 6 to 10 seconds before withdrawal to ensure full absorption of insulin, but this practice was found only in around one-third of patients in our study, which is lower than Bari et al. (39%) and Poudel et al. (53.5%) [29, 30]. Errors in the insulin injection technique also included not removing the needle cap and used needle from pen after injection (90%) and not mixing cloudy insulin (51.2%). The proper insulin injection technique is vital in glycemic control, and the incorrect injection technique may lead to poor absorption, thereby severe outcomes such as hypoglycemia, hypoglycemia, lipohypertrophy, or lipoatrophy [23, 31].

The present study found a significant increase in participants' knowledge of insulin pen use and injection practice over a two-month follow-up. The improvement remained significant after two months despite a slight decrease in the knowledge of insulin use one month after the intervention. Such improvement is confirmed in a cohort education study in Poland [32] where education intervention results in positive changes in many aspects of insulin use, patients' satisfaction, and blood glucose control. These findings emphasize the vital role of health education, especially continuous assessment and reeducation where healthcare staff can make necessary modification to health education plan for optimizing diabetes management. Moreover, we found that the levels of knowledge about hypoglycemia and insulin use were negatively associated with the increased age and education level. This finding is supported by results from previous studies [13, 33]. The finding implies that healthcare professionals should pay attention to those in this high-risk group in implementing health education.

In terms of treatment, in the current study, patients who had received a high frequency of counseling about insulin pen since their diagnosis were more likely to have good knowledge about hypoglycemia and insulin use. A possible explanation is that people feel difficult to recall what they learn only one time or forget the initial information provided. Therefore, regular education methods such as reeducation or teach-back are demanding during the course of T2DM treatment [28]. In our study, patients received health education at each visit during the follow-up, and thus, both the knowledge and practice remained high after two months. Counselors also have an important role in the changes of knowledge and practice among patients with T2DM. We found that the patients who had received counseling from pharmacists since their diagnosis had better awareness of insulin pen use compared with those who had received such support from nurses and doctors. Hughes, Wibowo, Sunderland, and Hoti (2017) also emphasized the role of pharmacists in diabetes care [34]. It is encouraging to increase the participation of pharmacists and interdisciplinary approaches in enhancing optimal T2DM treatment.

Several implications can be learned from this study. If the low level of good knowledge and practice is true, a large number of patients with T2DM may be at high risk of hypoglycemia and other complications. This indicates an urgent need for further intervention for this population. Moreover, this study has confirmed the previous finding and contributed the evidence of a positive effect of health education on study outcomes and potential factors. However, the application of this intervention requires further modification in clinical practice according to the types of hospital (community/general/university hospital), patients (inpatients/outpatients/patients with T1DM/T2DM), demographic, health-related, and counseling characteristics. This suggests that healthcare providers and healthcare professionals should provide specific interventions aiming to optimize the quality of life of patients with DM.

Findings from our study should be interpreted in light of several limitations. First, this study was conducted at only one primary care hospital in an urban area with a relatively small sample size. This may affect the generalizability of the sample. It is possible that in different settings such as rural or suburban, patients may react differently to the health educational intervention. Further research should include a larger sample size and target population in multiple and various kinds of clinical settings. Second, the relatively short follow-up over a two-month period may not be enough to observe the stability and the long-term effect of the intervention on the study outcomes. We reminded the participants about knowledge and practice at every visit during the study but were unable to know whether the patients have good knowledge and practice without such reminders after the study. Further studies are needed to investigate the intervention intensity needed for the patients to have good knowledge and practice for a long time. Lastly, although our questionnaire was based on current standards and guidelines, the reliability and validity of this questionnaire warrant further investigation. Validated scales to measure knowledge and practice toward hypoglycemia and insulin pen use are needed so that results can be compared across settings.

## 6. Conclusions

Patients with T2DM have a low level of knowledge and practice. Fortunately, the health education intervention is effective in improving knowledge and practice in this population. There is a pressing need for such intervention at primary care hospitals to optimize treatment for patients with T2DM, possibly focusing on those who had characteristics that have the best effectiveness found in this study.

## Figures and Tables

**Figure 1:**
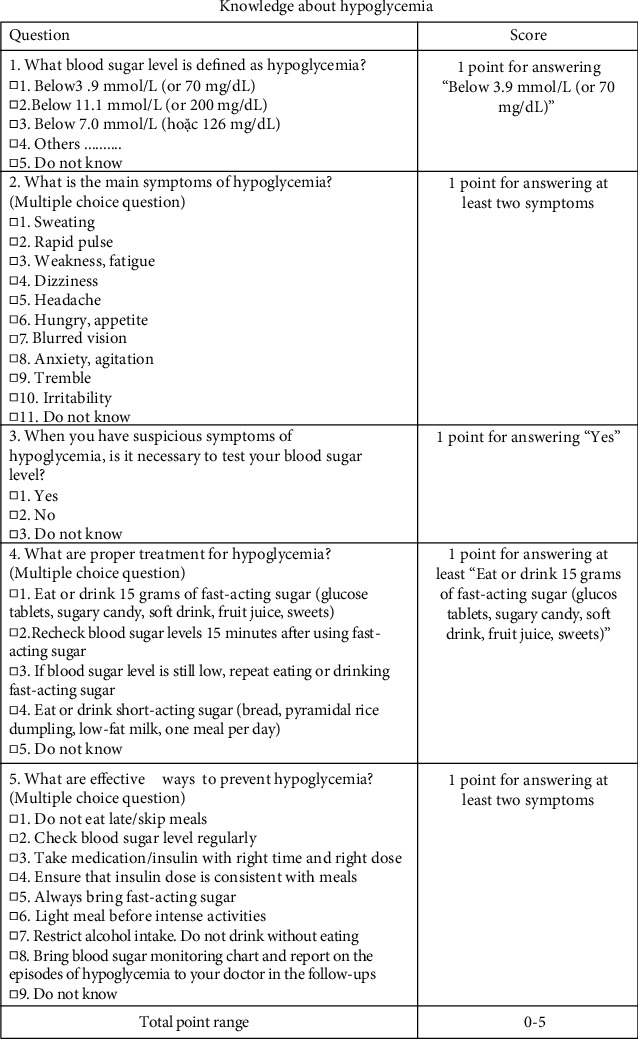
Knowledge about hypoglycemia.

**Figure 2:**
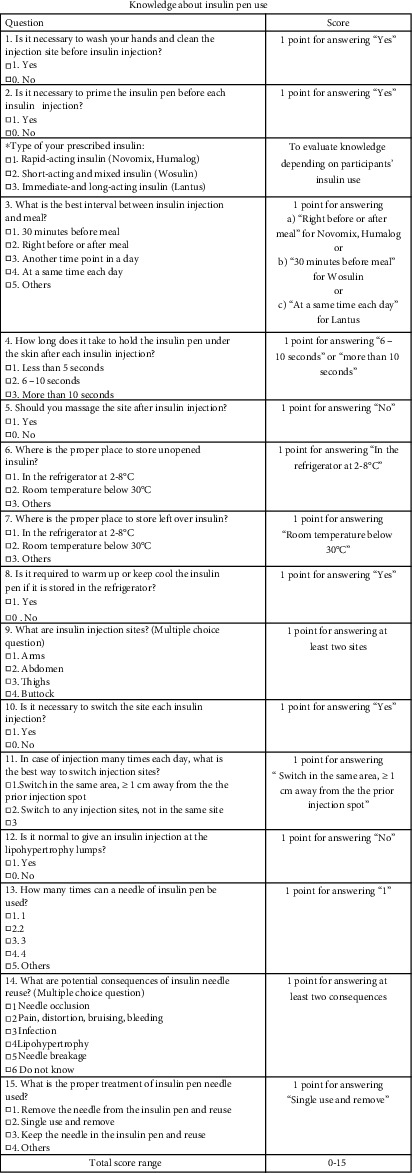
Knowledge about insulin pen use.

**Figure 3:**
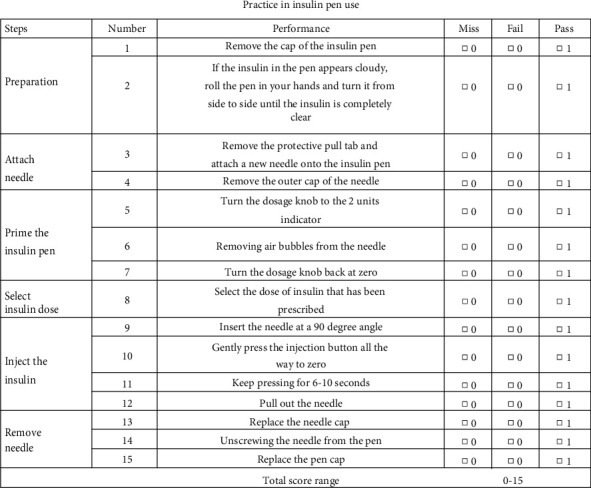
Practice in insulin pen use.

**Table 1:** Knowledge about hypoglycemia.

Good knowledge	Before intervention (*T*_0_) *n* (%)	Right after intervention (*T*_1_) *n* (%)	After one month (*T*_2_) *n* (%)	After two months (*T*_3_) *n* (%)	*P* (*T*_1_ vs. *T*_0_)	*P* (*T*_2_ vs. *T*_0_)	*P* (*T*_3_ vs. *T*_0_)
Hypoglycemia definition							
Yes	11 (13.8)	65 (81.3)	34 (42.5)	60 (84.5)	<0.001	<0.001	<0.001
No	69 (86.2)	15 (18.7)	46 (57.5)	11 (15.5)			

Hypoglycemia symptoms							
Yes	48 (60.0)	69 (86.3)	59 (73.8)	64 (90.1)	<0.001	0.003	<0.001
No	32 (40.0)	11 (13.7)	21 (26.2)	7 (9.9)			

Hypoglycemia testing							
Yes	24 (30.0)	70 (87.5)	56 (70.0)	67 (94.4)	<0.001	<0.001	<0.001
No	56 (70.0)	10 (12.5)	24 (30.0)	4 (5.6)			

Hypoglycemia treatment							
Yes	12 (15.0)	53 (66.3)	46 (57.5)	62 (87.3)	<0.001	<0.001	<0.001
No	68 (85.0)	27 (33.7)	34 (42.5)	9 (12.7)			

Hypoglycemia prevention							
Yes	9 (11.3)	33 (41.3)	40 (50.0)	57 (80.3)	<0.001	<0.001	<0.001
No	71 (88.7)	47 (58.7)	40 (50.0)	14 (19.7)			

Total score (median, IQR)	1 (0-2)	4 (3-4)	3 (2-4)	5 (4-5)	<0.001	<0.001	<0.001

**Table 2:** Knowledge about using insulin pen.

Good knowledge	Before intervention (*T*_0_) *n* (%)	Right after intervention (*T*_1_) *n* (%)	After one month (*T*_2_) *n* (%)	After two months (*T*_3_) *n* (%)	*P* (*T*_1_ vs. *T*_0_)	*P* (*T*_2_ vs. *T*_0_)	*P* (*T*_3_ vs. *T*_0_)
Hand washing and injection site cleaning							
Yes	63 (78.8)	80 (100)	80 (100)	71 (100)	<0.001	<0.001	0.0001
No	17 (21.2)	0 (0)	0 (0)	0 (0)			

Pushing the air bubble out before injection							
Yes	16 (20.0)	72 (90.0)	65 (81.2)	66 (93.0)	<0.001	<0.001	<0.001
No	64 (80.0)	8 (10.0)	15 (18.8)	5 (7.0)			

Injection time							
Yes	41 (51.3)	76 (95.0)	72 (90.0)	65 (91.6)	<0.001	<0.001	<0.001
No	39 (48.7)	4 (5.0)	8 (10.0)	6 (8.4)			

Duration of syringe under skin after injection							
Yes	34 (42.5)	78 (97.5)	68 (85.0)	71 (100)	<0.001	<0.001	<0.001
No	46 (57.5)	2 (2.5)	12 (15.0)	0 (0)			

Massage injection site							
Yes	47 (58.8)	73 (91.3)	70 (87.5)	70 (98.6)	<0.001	0.0001	<0.001
No	33 (41.2)	7 (8.7)	10 (12.5)	1 (1.4)			

Unused insulin storage							
Yes	69 (86.3)	78 (97.5)	73 (91.3)	71 (100)	0.004	0.219	0.004
No	11 (13.7)	2 (2.5)	7 (8.7)	0 (0)			

Leftover insulin storage							
Yes	21 (26.3)	70 (87.5)	57 (71.3)	65 (91.6)	<0.001	<0.001	<0.001
No	59 (73.7)	10 (12.5)	23 (28.7)	6 (8.4)			

Stabilize temperature before injection							
Yes	26 (32.5)	78 (97.5)	76 (95.0)	71 (100)	<0.001	<0.001	<0.001
No	54 (67.5)	2 (2.5)	4 (5.0)	0 (0)			

Injection site							
Yes	46 (57.5)	80 (100)	79 (98.8)	71 (100)	<0.001	<0.001	<0.001
No	34 (42.5)	0 (0)	1 (1.2)	0 (0)			

Changing injection site							
Yes	80 (100)	79 (98.8)	80 (100)	71 (100)	1.000	1.000	1.000
No	0 (0)	1 (1.2)	0 (0)	0 (0)			

Changing injection site every day							
Yes	28 (35.0)	65 (81.3)	58 (72.5)	61 (85.9)	<0.001	<0.001	<0.001
No	52 (65.0)	15 (18.7)	22 (27.5)	10 (14.1)			

Injection site selection							
Yes	73 (91.3)	78 (97.5)	77 (96.3)	71 (100)	0.180	0.344	0.016
No	7 (8.7)	2 (2.5)	3 (3.7)	0 (0)			

Number of injections per needle							
Yes	8 (10.0)	61 (76.3)	38 (47.5)	57 (80.3)	<0.001	<0.001	<0.001
No	72 (90.0)	19 (23.7)	42 (52.5)	14 (19.7)			

Consequences of reuse needle so many times							
Yes	9 (11.3)	55 (68.8)	49 (61.3)	58 (81.7)	<0.001	<0.001	<0.001
No	71 (88.7)	25 (31.2)	31 (38.7)	13 (18.3)			

Needle treatment after injection							
Yes	6 (7.5)	56 (70.0)	41 (51.3)	57 (80.3)	<0.001	<0.001	<0.001
No	74 (92.5)	24 (30.0)	39 (48.7)	14 (19.7)			

Total score (median and IQR)	7 (5-8)	14 (13-14.5)	12 (11-14)	14 (13-15)	<0.001	<0.001	<0.001

**Table 3:** Practice in insulin pen use.

Insulin pen use		Before intervention (*T*_0_) *n* (%)	Right after intervention (*T*_1_) *n* (%)	After one month (*T*_2_) *n* (%)	After two months (*T*_3_) *n* (%)	*P* (*T*_1_ vs. *T*_0_)	*P* (*T*_2_ vs. *T*_0_)	*P* (*T*_3_ vs. *T*_0_)
Preparation	Remove the cap of the insulin pen	80 (100)	80 (100)	80 (100)	71 (100)	1.000	1.000	1.000
	If the insulin in the pen appears cloudy, roll the pen in your hands and turn it from side to side until the insulin is completely clear.	39 (48.8)	63 (78.8)	57 (71.3)	64 (90.1)	<0.001	0.0005	<0.001

Attach needle	Remove the protective pull tab and attach a new needle onto the insulin pen	80 (100)	80 (100)	80 (100)	71 (100)	1.000	1.000	1.000
	Remove the outer cap of the needle	80 (100)	80 (100)	80 (100)	71 (100)	1.000	1.000	1.000

Prime the insulin pen	Turn the dosage knob to the 2 units indicator	11 (13.8)	70 (87.5)	55 (68.8)	63 (88.7)	<0.001	<0.001	<0.001
	Removing air bubbles from the needle	6 (7.5)	54 (67.5)	44 (55.0)	54 (76.1)	<0.001	<0.001	<0.001
	Turn the dosage knob back at zero	11 (13.8)	67 (83.8)	55 (68.8)	63 (88.7)	<0.001	<0.001	<0.001

Select insulin dose	Select the dose of insulin that has been prescribed	78 (97.5)	79 (98.8)	80 (100)	71 (100)	1.000	0.500	0.500

Inject the insulin	Insert the needle at a 90 degree angle	68 (85.0)	78 (97.5)	80 (100)	71 (100)	0.002	0.001	0.002
	Gently press the injection button all the way to zero	80 (100)	80 (100)	80 (100)	71 (100)	1.000	1.000	1.000
	Keep pressing for 6-10 seconds	27 (33.8)	62 (77.5)	57 (71.3)	68 (95.8)	<0.001	<0.001	<0.001
	Pull out the needle	80 (100)	80 (100)	80 (100)	71 (100)	1.000	1.000	1.000

Remove needle	Replace the needle cap	7 (8.8)	66 (82.5)	54 (67.5)	61 (85.9)	<0.001	<0.001	<0.001
	Unscrewing the needle from the pen	8 (10.0)	66 (82.5)	54 (67.5)	61 (85.9)	<0.001	<0.001	<0.001
	Replace the pen cap	80 (100)	80 (100)	80 (100)	71 (100)	1.000	1.000	1.000

Total score (median and IQR)		9 (8-10)	14 (12.5-15)	13 (11-15)	15 (13-15)	<0.001	<0.001	<0.001

**Table 4:** The association between patients' characteristics and overall knowledge and practice scores.

Characteristics	Score on knowledge about hypoglycemia		Score on knowledge about insulin pen		Score on practice using insulin pen	
	Coef (95% CI)	*P*	Coef (95% CI)	*P*	Coef (95% CI)	*P*
Demographics						
Ethnicity						
Kinh	Ref		Ref		Ref	
Hoa	-0.46 (-0.92; -0.01)	0.046	-0.85 (-1.50; -0.19)	0.011	-0.01 (-0.57; 0.56)	0.985
Age category (year)						
<50	Ref		Ref		Ref	
50-60	-0.65 (-1.10; -0.20)	0.005	-0.93 (-1.66; -0.19)	0.014	-0.69 (-1.37; -0.001)	0.050
>60	-0.71 (-1.12; -0.30)	0.001	-1.01 (-1.69; -0.33)	0.004	-1.43 (-2.11; -0.75)	**<**0.001
Sex						
Female	Ref		Ref		Ref	
Male	0.23 (-0.16; 0.62)	0.256	0.02 (-0.53; 0.58)	0.932	0.53 (-0.04; 1.09)	0.070
Education level						
Primary school	Ref		Ref		Ref	
Secondary school	0.42 (-0.04; -0.89)	0.074	0.62 (-0.06; 1.29)	0.072	0.06 (-0.68; 0.79)	0.882
High school or over	0.84 (0.43; 1.25)	<0.001	0.78 (0.28; 1.29)	0.002	0.50 (-0.09; 1.09)	0.094
Occupation						
Government employee	Ref		Ref		Ref	
Retired	-0.68 (-1.69; 0.34)	0.190	-0.33 (-2.34; 1.67)	0.745	-1.37 (-2.82; 0.08)	0.065
Others	-0.83 (-1.75; 0.09)	0.076	-0.69 (-2.61; 1.22)	0.480	-1.05 (-2.29; 0.19)	0.096
Average monthly income (million VND)						
<3	Ref		Ref		Ref	
3-<7	-0.05 (-0.48; 0.38)	0.829	0.31 (-0.30; 0.92)	0.315	0.51 (-0.10; 1.11)	0.101
≥7	0.75 (0.30; 1.19)	0.001	0.91 (0.38; 1.45)	0.001	1.20 (0.59; 1.82)	<0.001
Health status						
Duration of living with diabetes (year)						
<1	Ref		Ref		Ref	
1-6	0.31 (-0.96; 1.58)	0.628	0.23 (-1.16; 1.61)	0.747	-0.30 (-1.98; 1.39)	0.729
>6	0.53 (-0.68; 1.75)	0.388	0.36 (-0.95; 1.68)	0.587	-0.27 (-1.80; 1.25)	0.726
*Duration of using insulin pen (year)*						
<1	Ref		Ref		Ref	
1-6	0.18 (-0.26; 0.62)	0.424	0.11 (-0.48; 0.70)	0.709	0.16 (-0.46; 0.78)	0.604
>6	0.16 (-0.52; 0.83)	0.653	0.31 (-0.45; 1.06)	0.427	-0.03 (-0.89; 0.83)	0.951
Type of insulin pen						
Fast-acting insulin	Ref		Ref		Ref	
Intermediate-acting insulin	-0.49 (-0.96; -0.01)	**0.044**	-0.25 (-1.04; 0.54)	0.531	-0.03 (-0.96; 0.90)	0.956
Long-acting insulin	-0.65 (-1.39; 0.08)	0.080	-0.19 (-1.26; 0.87)	0.723	0.45 (-0.64; 1.54)	0.417
Number of insulin injections per day						
1	Ref		Ref		Ref	
2	0.03 (-0.65; 0.72)	0.922	-0.22 (-1.20; 0.76)	0.664	-0.52 (-1.29; 0.25)	0.182
3	0.10 (-0.57; 0.78)	0.764	-0.31 (-1.27; 0.65)	0.525	-0.74 (-2.15; 0.67)	0.303
Health counseling services received since your diagnosis						
Ever received counseling about insulin pen						
No	Ref		Ref		Ref	
Yes	-0.17 (-0.77; 0.43)	0.584	0.04 (-0.99; 1.06)	0.947	0.12 (-1.02; 1.25)	0.839
Received counseling about insulin pen from whom						
Pharmacist	Ref		Ref		Ref	
Nurse	0.15 (-0.45; 0.75)	0.630	-0.73 (-1.30; -0.17)	0.010	-0.11 (-0.78; 0.56)	0.746
Doctor	0.32 (-0.09; 0.75)	0.124	-0.94 (-1.29; -0.60)	<0.001	-0.40 (-0.88; 0.08)	0.102
Frequency of receiving counseling about insulin pen						
Only the first time when receiving insulin pen	Ref		Ref		Ref	
Several times during the treatment	1.73 (1.53; 1.94)	<0.001	0.26 (-0.001; 0.53)	0.051	1.11 (0.82; 1.39)	<0.001
The last time received counseling about insulin pen (year)						
<1	Ref		Ref		Ref	
1-5	0.20 (-0.31; 0.70)	0.446	0.25 (-0.37; 0.86)	0.427	0.14 (-0.52; 0.79)	0.681
>5	0.17 (-0.46; 0.80)	0.602	0.66 (-0.16; 1.47)	0.113	0.30 (-0.49; 1.09)	0.453

## Data Availability

The data used to support the findings of this study are restricted by the local ethics committee in order to protect patient privacy. Data are available from Truc Thanh Thai, email: thaithanhtruc@ump.edu.vn for researchers who meet the criteria for access to confidential data.
